# A Comparison of Mathematical Models for Polarization of Single Eukaryotic Cells in Response to Guided Cues

**DOI:** 10.1371/journal.pcbi.1001121

**Published:** 2011-04-28

**Authors:** Alexandra Jilkine, Leah Edelstein-Keshet

**Affiliations:** 1 Green Comprehensive Center for Computational and Systems Biology, Department of Pharmacology, University of Texas Southwestern Medical Center, Dallas, Texas, United States of America; 2 Mathematics Department, University of British Columbia, Vancouver, British Columbia, Canada; North Carolina State University, United States of America

## Abstract

Polarization, a primary step in the response of an individual eukaryotic cell to a spatial stimulus, has attracted numerous theoretical treatments complementing experimental studies in a variety of cell types. While the phenomenon itself is universal, details differ across cell types, and across classes of models that have been proposed. Most models address how symmetry breaking leads to polarization, some in abstract settings, others based on specific biochemistry. Here, we compare polarization in response to a stimulus (e.g., a chemoattractant) in cells typically used in experiments (yeast, amoebae, leukocytes, keratocytes, fibroblasts, and neurons), and, in parallel, responses of several prototypical models to typical stimulation protocols. We find that the diversity of cell behaviors is reflected by a diversity of models, and that some, but not all models, can account for amplification of stimulus, maintenance of polarity, adaptation, sensitivity to new signals, and robustness.

## Introduction

The ability to form a distinct front and back in response to chemical or mechanical stimuli is inherent in most eukaryotic cells (from yeast to neurons), and plays important roles in differentiation, development, and motility. Broadly speaking, *polarization* is a redistribution of multiple proteins and lipids in the cell. Some of these components include phosphoinositide lipids [1], PAR proteins [2], and Rho family GTPases [3]. Typically, certain proteins (Cdc42, Rac, PI3K, Par3/6) and lipids (PIP2/3) determine the cell front (anterior end) and others (Rho, PTEN) are common at the rear, though details vary from cell to cell. Many of these are conserved in polarization across a broad range of cell types.

Eukaryotic cells have spatial *gradient sensing* (unlike bacteria, which use a temporal mechanism), that is, they can detect concentration gradients as low as a few percent across the diameter of a cell [4]–[7]. These stimuli evoke macroscopic gradients of polarity proteins/lipids. Polarity is commonly studied in motile cells that undergo *chemotaxis* (movement in the direction of a chemical gradient). We focus this review on the response to stimuli such as chemoattractants cyclic AMP (cAMP), fMLP, and platelet-derived growth factor (PDGF). Motility is known to require localized assembly of actin filaments in the lamellipod, which forms the leading edge of a motile cell. However, polarization precedes motility, and occurs also in the absence of movement and in the absence of the cytoskeleton in many cell types.

Understanding the signaling cascades that link cell surface receptors to motility and chemotaxis is very challenging. For this reason, theorists have focused on smaller systems in an effort to understand how polarization is achieved. The underlying molecular network, akin to a wiring diagram of an electrical circuit, is then dissected into modules, each comprised of a few components. By understanding these modules, and then linking these together, we hope to understand the function of the molecular network as a whole [8], [9]. In a distinct approach, theorists askew the detailed network, and look at simpler models that have analogous capabilities (e.g., symmetry breaking, response to graded or noisy inputs, etc.). Here, we survey largely models of the latter type, and briefly mention a few of the former.

We first summarize collective and universal features of cell polarization. These lead to a number of important questions that theory has been directed at answering. We then briefly describe cell types commonly used to study polarity and indicate how their polarization behavior fits into the overall scheme. Next, we survey several classes of mathematical models proposed to explain how cell polarization occurs. To focus this review on main insights (rather than a multiplicity of details), we concentrate here on the qualitative aspects of the models, with occasional mention of biochemical correspondence. We devise a set of *in silico* tests that are based on common experimental protocols. This allows us to compare the performance of four typical models in a standardized approach. We argue that some classes of models are more appropriate for describing the behavior of certain cell types but miss important features of other cell types.

### Universal Features of Polarizing Cells

The following features of cell polarization are shared by many cell types.

1. Cells are able to sense both steep and shallow external gradients (where the difference between front and back receptor concentration is as small as 1%–2%) within a vast range of concentrations. Polarization leads to an *amplification* of this asymmetry to some macroscopic level.
2. Polarized chemotactic cells remain *sensitive to new stimuli*, and can reorient when the stimulus gradient is changed.
3. In many types of cells, polarity is maintained after the triggering stimulus is removed (*maintenance*). Some evidence suggests that this persistence requires an intact cytoskeleton.
4. Some cells *spontaneously polarize*, that is, they establish an axis of asymmetry in the absence of spatial cues.
5. Some cell types exhibit *adaptation* in a uniform stimulus, that is, the cells generate a persistent response to a gradient of chemoattractant, but transient response to a temporal change in a uniform stimulus.
6. In response to multiple stimuli (such as two sources of chemoattractant), some cells form multiple “fronts” in certain situations, whereas others rapidly resolve the conflict with a *unique axis of polarity*.
7. In some cells, pseudopods are continually extended and retracted. Some of these types of cells reorient by splitting one pseudopod into two, one of which becomes dominant.

Here, we have outlined the most prevalent observations. Other cell-type specific behaviors are discussed in the next section. Such observations have led to numerous theoretical questions. These include (but are not limited to) the following: (1) How is amplification created? What feedback loops are necessary so that stochastic fluctuations in the local concentrations of polarity factors are amplified into a single dominant asymmetry? (2) How is polarization maintained in a way that still allows sensitivity to new signals? (3) How do feedbacks from the actin and/or microtubule cytoskeleton enable cells to maintain their polarity? (4) How can spontaneous polarization be reconciled with adaptation to a uniform stimulus that is also observed under some conditions? (5) How do cells resolve multiple conflicting stimuli to establish a single ultimate “front” of activity?

## Polarity in Various Cell Types

It could be argued that motility and polarization in cells have been crafted by evolution in the context of distinct functions or environmental challenges. For example, cells of the amoeba *Dictyosteium discoideum* chemotax under starvation conditions, relaying signals to one another to form aggregates. Neurons extend processes over long distances (meters) following specific guidance cues to their synaptic targets. In contrast, yeast is nonmotile, reacting to mating pheromones by formation of a “schmoo-like” shape. The diversity of cell functions might suggest that many distinct underlying mechanisms are at play, so it is remarkable how universal are the conserved aspects of polarization. However, experimental science specializes on a limited number of cell types, and hence there tend to be many species-centric views of polarization. This tends to obscure both the common and universal features, as well as the distinct differences between cell types.

The budding yeast, *Saccharomyces cerevisiae*, provides a simple, genetically very well-understood nonmotile cell that exhibits several polarization responses, including maintenance **(3)**, spontaneous polarization **(4)**, and unique axis of polarity **(6)**. In yeast, polarization is required for mating and for budding (formation of a daughter cell) [10]. Mating is analogous to cell migration in that cells polarize and grow toward a gradient of mating pheromone. When exposed to uniform concentrations of pheromones, mating projections form in random orientations. During bud formation, Cdc42 concentrates in a “polar cap”, marking the site for a new daughter cell [11]. New buds usually form in a direction specified by a previous bud scar, but when bud site selection genes are genetically abrogated, the budding occurs at random locations [12]. Cdc42 accumulation on the cell membrane during bud formation is regulated by two parallel positive feedback loops. The first is a slow, actin-dependent loop and the second, a fast, actin-independent mechanism [13], [14]. If one loop is disabled, polarization occurs; if both are inhibited, no polarity is possible. In cells with actin-independent feedback only, polarization is delayed, but the resulting polar caps are stable. If just the actin-dependent loop is disabled, polar caps form quickly, but often drift or disappear [10]. It has been reported [15] that cells lacking the positioning factor Bud1 are unable to stably maintain the position of the polar cap at the onset of budding; this may indicate the presence of an additional negative feedback loop.

The chemotactic social amoeba, *Dictyostelium discoideum*, senses gradients of the chemoattractant cAMP, chemotaxes, and secretes cAMP to attract other amoebae under starvation conditions. Like yeast, *D. discoideum* can spontaneously polarize in the absence of a gradient [16]. Unlike mammalian cells that require a stimulus to initiate motility, in *D. discoideum*, pseudopods are continually formed, and take over dominance of the leading direction depending on perceived cues [17]. Unstimulated cells exhibit dynamical wave-like protrusions [18], [19]. Chemotactic reorientation is sometimes achieved by the splitting of pseudopods [20]. After a Y-shaped split, one branch becomes dominant while the other regresses, leading to sequential turns if the external cue is altered. Such splitting has also been observed in the absence of external cues [21]. Essentially, *D. discoideum* has polarity attributes **(1–5,7)** as described above. *D. discoideum* cells with an immobilized actin cytoskeleton (after treatment with the drug latrunculin) form multiple areas of localized PIP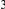 activity [22]. The cell's internal chemical polarization shows amplification of the external gradient up to 7-fold over a wide range of concentrations [22]. These cells easily repolarize when the gradient direction is changed and adapt to uniform elevation of chemoattractant [23], [24]. Latrunculin-treated *D. discoideum* cells are seen to respond to and to amplify external signal asymmetry and adapt to uniform stimuli [22], [25], demonstrating that gradient sensing can be decoupled from the cytoskeleton and the resulting morphological polarity [26]. Because *D. discoideum* has a small haploid genome, genetic manipulations are easy to perform. As a result, the molecular details of how the directional sensing system operates in *D. discoideum* are much better understood than in most mammalian chemotactic cells [27]. In mammalian cells, Cdc42 and Rho GTPases play an important role in polarity establishment, while in *D. discoideum*, genes homologous to Rho or Cdc42 have not been discovered [28], so other components are likely to be involved in actin and myosin regulation in these cells [29].

Mammalian neutrophils (white blood cells), like those of *D. discoideum*, have highly sensitive gradient sensing and strong internal amplification. These immune system cells migrate directionally in response to external N-formylated peptide gradients produced by bacteria. These cells exhibit features **(1–4)** and **(6)**. However, unlike *D. discoideum* cells, the neutrophil default state is nonmotile, and neutrophils spontaneously polarize only if chemoattractant is present. Wild-type neutrophils have a unique axis of polarity; stimulating human neutrophils with spatially homogeneous chemoattractant induces ruffles that consolidate into a single pseudopod within minutes [30]. However, cells where RhoA has been inhibited [31], or where the lipid domains in the plasma membrane have been chemically altered [32], can form multiple protrusions in a uniform field of chemoattractant. Fluorescent probes have revealed front-localizing (phosphoinositide lipids PIP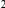 and PIP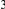, F-actin, and Rho GTPases Cdc42 and Rac), versus back-localizing signaling components (RhoA and myosin II) [31], [33]. In neutrophils, but not *D. discoideum*, there is evidence for front–back mutual inhibition (e.g., by Cdc42 and Rho) [31]. This lends support to the idea that cell polarization is a self-organizing mechanism that emerges as a result of feedback loops between various polarity factors [34]. Several positive feedback circuits for actin accumulation involving phosphoinositides have been found in chemotactic cells [35]. Latrunculin treatment inhibits actin polymerization, disabling these loops.

Fibroblasts are connective tissue mammalian cells responsible for wound healing. These cells migrate in response to gradients of platelet-derived, epidermal, and other growth factors. These cells have attributes **(1,2)**, **(4)**, and **(5)**. However, PDGF sensing requires steeper gradients and a much narrower range of absolute chemoattractant concentrations than neutrophils and *D. discoideum* [36]. Fibroblasts are larger than neutrophils and *D. discoideum* (50–150 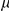
m versus 10–20 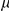
m), with a more complex cytoskeleton, and, unlike the latter, require an intact microtubule (MT) cytoskeleton for migration. Fibroblasts polarize spontaneously on adhesive substrates (e.g., fibronectin or poly-D-lysine), on the timescale of 30–50 min [37], involving cell spreading mediated by integrins. Their motility is much slower than that of neutrophils (2 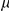
m/min compared to 20 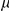
m/min), likely due to enhanced adhesivity. As in other migrating cells, fibroblasts transduce external gradients to the actin cytoskeleton via PIP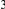 and Rho GTPase signaling [38], but a positive feedback loop involving actin and PIP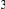 in neutrophils [39] has not been observed in fibroblasts [36], [37].

Nerve cells have to extend long distances in accurate migration during development, demonstrating features **(1–3,5)** and **(7)**. This extension is mediated by growth cones, flat lamellipodial-like structures that sense and respond to the environment, much as the motile cells described above do. In neuronal growth cones, a guidance response can be detected in gradients as shallow as 1%. Responding to a variety of diffusible and adhesive chemoattractants and repellents, growth cones adapt their sensitivity to a broad range of concentrations encountered over their route. Cytoskeletal regulation in the extending growth cone is governed by many of the same molecular components mentioned above, including Rho GTPases, PI3K, Ena/VASP, and cofilin [40]. Formation of “front-like” and “rear-like” portions of the growth cone, as well as turning responses and biased branch selection, resemble analogous behavior of the previously described motile cells [41], [42].

Other model systems for cell migration include keratocytes, fast-moving epithelial cells from scales of fish, that exhibit features **(2–4)** and **(6)**. Unlike other motile cells, keratocytes maintain a consistent shape and smooth gliding motion during motility [43], and react (and reorient) to mechanical, rather than chemical, stimuli. Fragments of keratocytes containing no nucleus or organelles exhibit polarization that is sustained long after the stimulus is gone [43]. These cells also spontaneously polarize upon separation from tissue by breaking symmetry in the actomyosin network at the rear of the cell [44].

Cell type differences described above are summarized in Table 1. We observe that even if cells share common polarity phenotypes, there are significant differences in how they come about. *D. discoideum* and neutrophils can sense very small gradients over a large range of concentrations, while fibroblasts operate in a much narrower concentration regime. Neutrophils polarize spontaneously only when exposed to stimulus, but *D. discoideum* polarizes even in the absence of cAMP. Gradient sensing in F-actin-inhibited *D. discoideum* cells is a transient phenomenon [22], while wild-type *D. discoideum* will migrate long distances in the absence of a gradient. Fibroblasts spontaneously polarize after being put on adhesive substrate, while keratocytes polarize after being detached from surrounding cells. The temporal aspects of polarity also differ, with neutrophils, *D. discoideum*, and keratocytes polarizing very fast (in less than a minute), while fibroblasts are much slower. While many of the same components are required for polarity in many cell types, again, there are differences. For instance, an intact MT cytoskeleton is required for migration of cultured fibroblasts and neurons, but not for keratocytes, neutrophils, and lymphocytes. The actin (but not the MT) cytoskeleton is involved in polarity establishment in budding yeast, while in fission yeast the opposite situation is true.

**Table 1:** Summary of cell type–specific polarization differences.

Cell type	Polarization Behaviors	Scale	Feedback Loops	Stimulus	Cytoskeleton
Budding yeast	Spontaneous polarization, unique axis of polarity	Size: 5 *µ*m, TP: 3 min	Cdc42 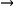 Cdc24 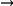 Cdc42,Cdc42 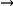 actin 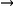 Cdc42	Bud1	Actin (MO)
*D. discoideum*	Gradient sensing (1% and up), adaptation (Lat), spontaneous polarization, high amplification, reorientation, maintenance, multiple fronts (Lat), unique axis (WT)	Size: 10–20 *µ*m, TP: 30–60 s, speed: 3–15 *µ*m/min	Amplification upstream of PI3K	cAMP	Actin (MO)
Fibroblasts	Gradient sensing, reorientation	Size: 50–150 *µ*m, TP: 30–50 min, speed: 1 *µ*m/min	Cdc42 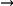 Rac 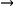 RhoA	PDGF, fibronectin, interleukins	Actin, MT, FA
Keratocytes	Spontaneous polarization, maintenance	Size: 10 *µ*m (fragments), 30–40 *µ*m (cells), speed: 10–40 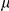 m/min,		Mechanical	Actin
Neutrophils	Gradient sensing, spontaneous polarization, high amplification, reorientation, unique axis (WT)	Size: 10 *µ*m, TP: 30 s, speed: 10–20 *µ*m/min	Front/back mutual inhibitionPIP3→actin→PIP3	fMLP, interleukins, others	Actin
Neurons	Attractive/repulsive turning, gradient detection, adaptation		Rac/Rho mutual inhibition	Netrins, semaphorins, ephrins	Actin, MT

FA, focal adhesions; Lat, latrunculin (no cytoskeleton); MT, microtubules; MO, maintenance only; TP, time to polarize' WT, wild-type.

Common experimental designs in polarity experiments include exposure to one or more localized stimuli, for example, pipette(s) of chemoattractant (fMLP for neutrophils, cAMP for *D. discoideum*, and EGF for fibroblasts), exposing cells to a gradient of chemoattractant in a microfluidic chamber, changing the location of the pipette or reversing the gradient to gauge the sensitivity to changing stimuli, and placing a cell in a well-stirred solution, i.e., uniform field of chemoattractant with stochastic fluctuations. In what follows, we devise a set of protocols based on such experimental tests and use these for testing the responses of a variety of models.

## A Survey of Mathematical Models

Here, we review several classes of mathematical models that have been proposed for individual cell polarization and list the features that these models were designed to explain. Not all polarization features occur in all cell types, and no single model addresses all of these questions, nor is it desirable to so construct such models, since, as discussed above, real cells of any given type display some, but not all of these properties. We summarize the strengths and weaknesses of each class of model in Table 2. In Table 3, we provide examples of published models that fit into these classes. For discussion of quantitative models of the cell signaling pathways involved in chemotaxis, see recent reviews [45], [46].

**Table 2:** Features of polarity explained by various classes of RD models.

Behavior	“Turing Type”	Wave-Based	Gradient Sensing
Maintenance of polarity	Yes	Yes	No
Multi-stimuli response	Yes (transient)	Yes (long time-scale)	Yes
High amplification	Yes	Yes	No
Adaptation	No	No	Yes
Spontaneous polarization	Yes	Yes	No
Reversible asymmetry	No	Yes	Yes

**Table 3:** Summary of published mathematical models for cell polarity.

Model	Class	Cell Type	Major Components
[79]	Stochastic	Budding yeast	Cdc42
[72]	Wave-based	*D. discoideum*	Phosphoinositides
[83], [84]	Stochastic	Chemotactic cells	Pseudopods
[78]	Wave-based	Chemotactic cells	Activator/inhibitor
[85]	Detailed biochemical	Neuron	Receptors, kinases, calcium channels, G-proteins
[99]	Turing type	Fission yeast	Activator-inhibitor
[73]–[76]	Detailed biochemical, wave-based	Chemotactic cells	Phosphoinositides, Rho GTPases,Actin, Arp2/3
[81], [82]	Detailed biochemical, stochastic	Chemotactic cells	Phosphoinositides
[54]	Detailed biochemical, Turing type	Budding yeast	Cdc42, Cdc24, Bem1, GAPs, GDI
[70]	Excitable system	*D. discoideum*	Activator/inhibitor
[100]–[102]	Gradient-sensing	Fibroblasts	Phosphoinositides
[61], [62], [64], [71], [103], [104]	Gradient-sensing	*D. discoideum*	Phosphoinositides
[63]	Gradient-sensing	*D. discoideum*	Activator/inhibitor
[105]	Turing type	Autocrine cells	EGFR
[106]	Gradient-sensing	*D. discoideum*	Phosphoinositides
[56], [88], [107]		Yeast	Cdc42
[50]	Turing type	Chemotactic cells	Activator-inhibitor
[51], [58]	Turing type	Chemotactic cells	Phosphoinositides
[52]	Turing type	Neutrophils	Activator-inhibitor
[86]	Detailed biochemical, wave-based	Neutrophils	Receptor, Ras, Rho, phosphoinositides, actin, myosin
[90]	Stochastic	Neutrophils	Receptors, inhibitors, mediators, microtubules
[53]	Turing type	Neutrophils	Rho GTPases
[15]	Wave-based	Yeast	Cdc42, Bem1
[108]	Gradient-sensing	*D. discoideum* and neutrophils	Second messenger
[109]		Neurons	Rho GTPases
[110]	Gradient-sensing	Chemotactic cells	Phosphoinositides

As discussed below, many models in the literature contain features that, broadly speaking, represent feedback in the form of activation, inhibition, depletion of a substrate, or combinations thereof. We have chosen to avoid grouping models simply by these categories. Instead, we discuss classes of dynamic behaviors, for example, models with instability to spatial noise, models supporting wave behavior, etc. (This also means that some models could fit into several classes.)

Because polarization involves chemical redistribution and symmetry breaking along one axis (“front to back”), it is often modelled by reaction diffusion (RD) systems in 1-D. Two common approaches are used. The first is to take a thin slice across the diameter of a cell with impermeable ends at opposite edges. (In that case, a polar chemical pattern has high level at one end, low at the opposite end.) Alternatively, some models describe chemical distributions only along the cell perimeter, and consider the “interior” as spatially uniform. (A polar pattern would then be a chemical distribution with one peak anywhere on the domain. In this way, even 1-D models can be used to account for, for example, multiple pseudopods around the perimeter of the cell.)

### Models with “Turing-Type” Pattern Formation

**Figure 1:**
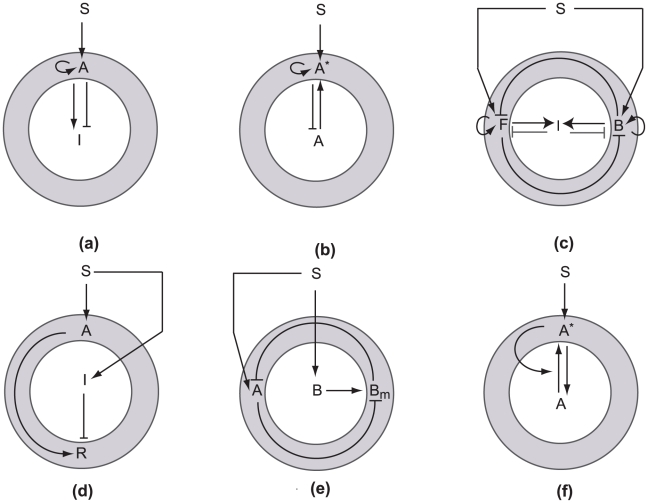
Schematic diagrams for proposed cell polarity mechanisms. Slow-diffusing (local) components are shown on the “cell membrane” (shaded), while fast-diffusing (global) components are shown in the interior of the cell (not to scale). *S*, signal; *A*, activator; *I*, inhibitor (unless otherwise indicated). (a) Model with a short-range activator and long-range inhibitor. See [48], [50]. (b) Model with substrate depletion. See [53], [54]. (c) A three-component model based on mutual inhibition [52]. *F* and *B* mutually inhibit each other and activate the global inhibitor. (Note: models (a–c) have Turing instabilities and we refer to these as “Turing-type” models.) (d) Local excitation, global inhibition (LEGI) [61]. The signal has identical effect on *A* and *I*, which together regulate a downstream response element (*R*). (e) Balanced inactivation mechanism [63]. *S* activates *A* and *B*, which produces *B_m_*. *B_m_* and *A* are mutual inhibitors. (f) The wave-pinning mechanism [76]. *S* affects a local membrane-bound activator (*A* ^*^), which is produced autocatalytically from its cytosolic substrate (*A*).

Turing [47] described the possibility of spatial instability in reaction-diffusion systems and argued that morphogenetic spatial patterns could be so created. His calculations were restricted to linear stability analysis. The destabilization of a uniform chemical distribution to spatial patterns at a given (range of) wave number is now commonly termed “Turing-type pattern formation” (or Turing bifurcation) in the mathematics community. Using simulations, Gierer and Meinhardt [48] showed that chemical interactions that depict local self-enhancement and long-range inhibition could produce biologically realistic patterns. Meinhardt and Gierer [49] were the first to point out the application of the idea of “lateral inhibition” to chemotactic orientation. While at the time, quantification of cell polarization biochemistry was still far into the future, this paper paved the way decades ahead of others. More recently, a paper by Meinhardt [50] rekindled this direction and initiated a new interest in modeling the mechanisms underlying chemotaxis. This has been followed in recent years by other models based on related theoretical foundations [51]–[54]. Recall that if a Turing instability is present, an unpolarized cell would react to any spatially varying stimulus (small or large) by breaking symmetry and forming a chemical pattern (see [55] for a review). Polarity requires that the dominant pattern have a single global maximum, though Turing patterns are notorious for producing multiple peak patterns under suitable ranges of parameters. The basic idea behind such diffusion-driven pattern formation rests in having processes with vastly different spatial characteristics that promote local activation and long-range inhibition. In two-component systems used to model polarization, this is achieved by postulating a large, membrane-bound autocatalytic “activator” with slow diffusion, and a small cytosolic inhibitor with faster, possibly infinite, diffusion, and hence more global reach (Figure 1a). Variations on this theme include a spatially uniform average for the cytosolic inhibitor [50], [56] or two mutually inhibitory activators (since double negative feedback is mathematically equivalent to autocatalysis [57]). Alternatively, the inhibitor can be replaced by substrate-depletion, which damps the activator production (Figure 1b).

A brief survey of polarization models with Turing instability includes the following: Meinhardt [50] proposed that two antagonists are necessary to achieve dynamic pseudopod extension; a global one to generate patterns and a local one to deactivate local maxima after some time. His model was able to account for generation and decay of local maxima, strong amplification, and rapid adaptation to a changed external gradient, as well as patterning and the persistence of that patterning in the absence of external signals. The model can be adapted to include a rest state. In Narang and co-workers [51], [58], the activator is associated with membrane phophoinositides, and cytosolic inositides are identified as the substrate. Narang [52] demonstrated that a Turing instability is impossible in a two-component mutually inhibiting system (which is equivalent to a single positive feedback loop), and derived a minimal three-component model consisting of two mutually inhibiting local activators that promote the synthesis of a diffusible inhibitor that, in turn, inhibits both the activators (Figure 1c). Otsuji et al. [53] proposed a mass-conserved activator-substrate system and showed that multi-peak states arise transiently, and are then replaced by a polar distribution. (Similar “winner-take-all” behavior is also seen in some other Turing-type models [51], [54], [58], [59].) Goryachev and Pokhilko [54] presented a detailed eight-variable model (for budding yeast) based on GTP-GDP cycling of Cdc42, its activator Cdc24, and the effector Bem1, and reduced it to a two-component activator-substrate system with similar essential features. In both [51], [58] and [54], the total amount of polarization readout (phosphoinositides in [51], [58]; Cdc42 in [54]) in membrane and cytosol is conserved.

Turing-type models are attractive theoretically, as they can account for spontaneous polarization, achieve a high degree of amplification, and maintain the polar pattern after the signal is removed. In *D. discoideum*, several pseudopods can coexist transiently. These could be considered multiple peaks of activity, but such peaks do not have a specific spacing behavior and are thus unlike a Turing pattern. In the two-antagonist model by Meinhardt [50], several maxima with irregular spacing can temporarily coexist. At the same time, some features of Turing-type models are less desirable. First, they fail to account for a resting nonpolar state in unstimulated cells (such as neutrophils)—every spatial disturbance, no matter how weak, breaks symmetry in the Turing regime. Second, and as mentioned above, multi-peak solutions can and do occur even in cases where these may not have biological relevance (e.g., in growing domains, or given appropriate variation of underlying parameters). Finally, the pattern, once formed, tends to “freeze”, and to be unresponsive to further stimuli [60]. This is fortuitous in some cases (e.g., formation of the Cdc42 cap in budding yeast, which is irreversible), but certainly not appropriate for migrating cells that have to respond to a highly variable or complex environment. To alleviate this problem, additional mechanisms or components are required to unfreeze the pattern; for example, Meinhardt and Gierer [49] showed that reorientation is possible if the system oscillates and that oscillation occurs if the inhibitor has a longer half-life than the activator. See also Meinhardt's local inhibitor [50], described above.

### Gradient-Sensing (Adaptation) Models

Early models for gradient sensing that were highly influenced by *D. discoideum* sought to account for both its adaptation to spatially uniform stimuli and its sensitivity to gradients. Levchenko and Iglesias [61] proposed a local excitation, global inhibition (LEGI) model consisting of a fast-acting local activator and a slow global inhibitor, both activated in direct proportion to external spatio-temporal stimuli (Figure 1d). By assuming that the response depends on the ratio of activator to inhibitor, they achieved perfect adaptation to uniform stimulation in a simple way that is robust to changes in parameters. LEGI accounts for some features of phospholipid PIP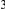 in latrunculin-treated *D. discoideum* cells [26], [60]–[62], such as an increasing response to stronger gradients, and a reversal of polarity if the gradient is reversed. This led its originators to propose that the roles of activator and inhibitor are played by PI3K, the kinase that synthesizes PIP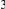, and PTEN, the phosphatase that reverses that reaction [61], motivating experimental investigation of the idea. Indeed, models with two coupled LEGI mechanisms were shown to mimic PI3K data in *D. discoideum* quite well [62], leading to rapid surge in the popularity of the LEGI mechanism throughout many cell biology papers. In a variant of LEGI, the “balanced inactivation” model [63] (Figure 1e) proposes that the cytosolic inhibitor converts to a membrane-bound form, inactivates the slow activator, and is thereby depleted. The addition of this extra component allows a switch-like response to external gradients, leading to a well-defined front and back.

The positive features of a LEGI mechanism (adaptation, sensitivity, response in proportion to signal appropriate response to multiple stimuli, concordance with latrunculin-treated *D. discoideum* data) come with some limitations. The LEGI mechanism by itself does not significantly amplify the external gradient, requiring additional mechanisms to do so [61], [64]. Interactions of a LEGI-type gradient sensing with existing asymmetries was studied in [64]. Further, on its own, LEGI cannot account for persistence of polarization: when the signal gradient is removed, the polar pattern disappears. In short, LEGI mechanisms as proposed in these references lack inherent pattern-formation capability. Thus, while LEGI accounts very well for some data, additional mechanisms (either up or downstream), possibly involving modules coupled to LEGI, would have to account for the observed persistent polarity of cells such as neutrophils and keratocytes [65]. As a final caution, recent knockout experiments in *D. discoideum* have revealed that mutants lacking PI3K can still polarize and chemotax [66], casting doubts on the suggested molecular identities ascribed to the components in [61], [62]. And so, the actual identities of the hypothesized LEGI activator and inhibitor are as yet unknown.

### Excitable Network and Wave-Based Models

A number of models for polarization are predicated on the idea that polarization is an outcome of a wave of activation (rather than growth of a unimodal pattern). In fact, some waves of activity (of actin polymerization [67], of the actin-regulator Hem1 [68]) are observed in cells. Furthermore, even if signaling is abolished due to the loss of receptors, localized regions of high concentrations of PIP3 and actin activity continue to emerge and disappear [69]. These are closely related to the subsequent formation of pseudopods. (The two-antagonist model discussed earlier [50] has been proposed to explain these dynamic features.) It has also been proposed [70], [71] that a spatially extended excitable system with a noisy input can likewise explain these types of phenomena. In the case of a rapidly diffusing activator, and a slower inhibitory process, a localized excitation will spread throughout the cell as a pulse/wave [68], [72]. When a local activator and a fast spreading inhibitor are considered in an excitable system, noise induces a spatiotemporal localized excursion to the excited state, and transient formation of localized patches is observed to recur [70]. When a chemical gradient is superimposed on the noise, a single patch tends to form in the direction of the gradient [71]. (An anonymous reviewer of this paper pointed out a link between [71] and [49] and added the following comments: (1) An oscillating activator-inhibitor system is sensitive to an external stimulus only in a narrow time window, just before the autocatalytic burst. (2) Due to the short period of time when competition between antipodal peaks can take place (during the burst), there is frequent formation of simultaneous peaks at opposite poles in the cell, leading to failure of polarization. (3) After a burst of global inhibitor, the refractory period would exclude other waves or peaks, in contrary to some observed behavior cell behavior [69]. See [49] for a discussion of oscillating activator–inhibitor distributions.)

It has also been shown that an activation wave triggered at one edge of a cell can produce a robust stationary polar pattern [73]–[76]. To do so, that wave has to stop inside the domain, creating a macroscopic difference between the level of activity at opposite cell edges. Necessary conditions for such wave-pinning (WP) behavior have been described in connection with Rho GTPase biochemistry [76] as follows: (1) The active GTP form, known to be bound to the cell membrane, and thus slowly diffusing, has to have positive feedback on its activation. (2) The inactive GDP form (known to associate with GDP dissociation inhibitor (GDI) to form a cytosolic, i.e., fast-diffusing complex) has to be depleted as activation takes place. In fact, this depletion halts the wave, and is thus essential for polarization. The latter property can be assured if the total amount of (active+inactive) protein is conserved. While mass conservation is shared with other models, the latter lack other features and fail to display stalling waves, for example, [53]. Similarly, other substrate-depletion models with saturating feedback terms look superficially similar to [76], but lack the features (conservation, bistability) required for such WP dynamics. The fact that WP in its simplest variant requires bistable kinetics [76] recalls models for protein-kinase cascades [77] where fast signaling exploits the traveling wave behavior. Unlike those, WP couples fast signaling with robust polarity.

WP models have a number of promising features. Unlike Turing-instability models, WP admits rest states that are stable to small amplitude stimuli. Thus, WP can account for resting cells that are unpolarized (e.g., neutrophils and keratocytes). A further advantage is that WP responds rapidly once the stimulus magnitude exceeds some threshold: in contrast to pattern formation close to a Turing bifurcation (which tends to slowly grow in magnitude from some small disturbance), WP forms a macroscopic peak of activity rapidly in response to a stimulus and then spreads to adjoining areas with significant speed. (A comparison of pattern-forming time scales in WP and Turing instability models is described in [76].) Unlike Turing patterns, the polarized stalled wave front in the WP model does not freeze: it can be reversed in response to new (sufficiently strong) stimuli. Like all models, WP has a number of drawbacks: first, the magnitude of the response (e.g., activity level at the front) is not directly proportional to the strength of the stimulus. Second, while patterns with multiple peaks are unstable, should they form due to noisy or competing stimuli, they can persist over long timescales until resolved. The presence of additional components can also accelerate the resolution of multiple peaks, as shown in [75]. As before, this suggests that WP on its own is not enough to account for all cell polarization features.

### Coupling Different Polarity Mechanisms

This review of individual models indicates that, while each one has good and bad aspects, none of the simple theoretical models accounts for all the features of polarity. This suggests hybrids where the output of one module is input to another. For example, WP might serve as a “symmetry breaking” module in larger models with other modules such as LEGI. An idea of coupling an adaptation module with a bistable system is discussed in [78] where LEGI is coupled to a bistable switch. Recently, coupling a LEGI module to an excitable network has been proposed to explain spontaneous spots of activity without stimulation [71].

WP and Turing instability mechanisms need not be mutually exclusive. The interactions between multiple components can result in a model that has WP behavior, but can also undergo a Turing instability in some parameter regimes [74]. This allows both sensitivity to small gradients, and an ability to reorient to new stimulus.

### Stochastic Models

While not the focus of this review, we briefly mention stochastic effects. Altschuler et al. [79] proposed a stochastic model based on positive feedback with mass action kinetics to explain spontaneous polarization in latrunculin-treated yeast. If, on average, a particle on the membrane recruits more than one particle from the cytosol during its residence time, aggregation can take place. In the stochastic regime this positive feedback, coupled with slow diffusion on the membrane, leads to recurrent patches of active particles on the membrane. If there are a lot of particles in the system, then positive feedback leads to activation all over the membrane, so that polarity is lost. Hence, this model predicts that polarity cannot occur for high numbers of molecules. By contrast, stochastic simulation of WP [80] showed that low copy number of reactant molecules fails to polarize the cell, whereas the probability of polarization increases, and approaches the behavior predicted by partial differential equations as the number of molecules increases. (The speed of polarization was the same in the stochastic and deterministic versions.)

A stochastic mechanism for gradient sensing based on phase separation (patch coalescence) was proposed by Gamba and coworkers [81], [82]. In that stochastic model, patches of PIP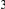 accumulate on the side of the spherical domain with higher concentration of activated receptors, and a coarsening process occurs with smaller patches eventually being absorbed by larger patches. However, the timescale on which polarization factors segregate into separated phases (representing spontaneous polarization) is about 100 minutes, which is rather slow for the timescale of polarization in many cell types.

Models of chemotaxis based on right-left splitting (or biased turning) of an existing pseudopod include [83], [84]. These account for both random motion and the biased random walk that migrating cells perform in the presence of shallow gradient, but do not depict the localization of polarized components within the cell.

### Detailed Biochemistry-Based Models

Many of the models mentioned above are “capability” models that describe theoretical mechanisms for cell polarity establishment. While such models are conceptually important, it is also useful to build detailed biochemical models “from the bottom up” that are based on identifiable molecular components and experimental findings [55]. Several such models already exist. Goryachev and Pokhilko [54] model Cdc42 and many of its activators and effectors in yeast polarity. Causin et al. [85] include many of the polarity players in neuronal polarity. Marée et al. [73] and Dawes and Edelstein-Keshet [75] simulate the interactions of Rho GTPases, phosphoinositides, and the actin cytoskeleton in a motile cell. Onsum and Rao [86] include some of the same molecules (e.g., phosphoinositides, actin, and the GTPase Ras, but not Rho GTPases) in neutrophil chemotaxis. However, it is difficult to directly compare these models as they are often tailored to specific cell types and conditions, and focus on properties of some specific subset of polarity regulators and experimental findings. Furthermore, the appreciation of the roles of regulatory substances changes rapidly. As an example, the phosphoinositide PIP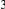 is no longer viewed as essential for chemotaxis under some conditions [66]. By understanding the necessary features for the types of phenomenological models discussed in this review, we can categorize the more complicated biochemistry-based models by the types of modules they contain (Turing instability, adaptation, wave-based, etc.) and identify the necessary components that give rise to their global behavior.

## A Comparison of Models

**Figure 2:**
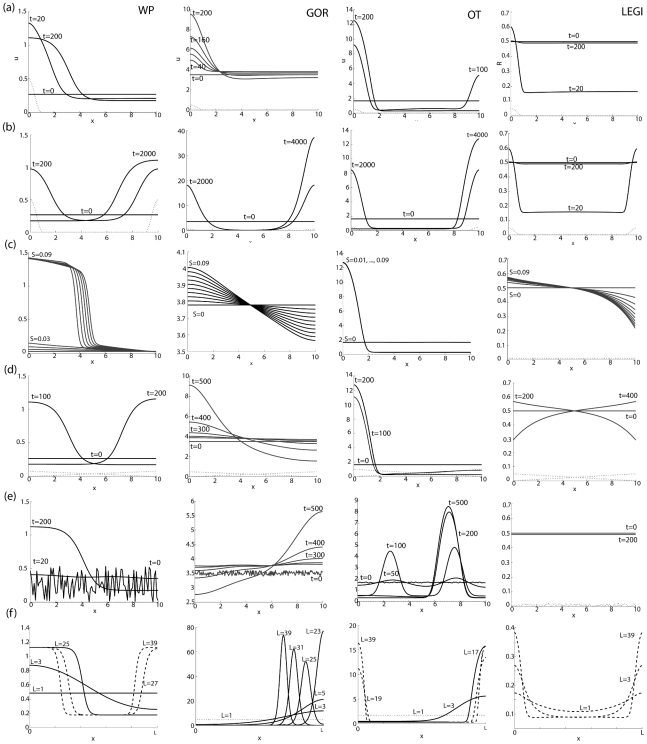
Comparison of polarization behavior of four models. Columns: (left to right) the wave-pinning (WP) system (2) [76], Goryachev's (GOR) system (4) [54], the Otsuji (OT) system (3) [53], and the LEGI system (5) [61]. Rows: the stimuli used: (a) single localized stimulus, (b) two competing local stimuli at opposite ends of the cell, (c) persistent graded stimuli of various strengths, (d) graded stimulus and its reversal, (e) noisy initial conditions, (f) increase in cell size. (See Methods for details.)

We chose typical representatives from the deterministic classes of models described above and subjected each of them to a set of common “stimulation protocols” (details in Methods). The representative models (columns in Figure 2) comprise (i) the WP [76] (wave-based) model, (ii) Goryachev's [54] (GOR) reduced Turing-type model, (iii) Otsuji's (OT) [53] Turing-type model, and (iv) the LEGI model [61]. Several of these models (WP, OT, GOR) are based on the biology of membrane-cytosol exchange of a pair of active/inactive Rho GTPase forms, making them good candidates for comparing proposed GTPase-based mechanisms for achieving polarity. As mentioned above, the LEGI model represents an unspecified activator, inhibitor, response element, and external stimulus [61].

The stimuli are: **(a)** transient-localized stimulus on the left edge, **(b)** two (identical) transient stimuli, one on each edge, **(c)** persistent graded stimulus, **(d)** transient gradient with reversal, **(e)** noisy initial conditions, and **(f)** change in cell size (see Methods). Except for case (e), we assume that the the initial conditions are spatially uniform for all models. In order to compare the speed of response, effect of cell size, etc., all models were calibrated to a timescale of seconds and length scale of microns. Responses of the four models are shown in rows of Figure 2 for a 10-*µ*m diameter cell, over a timespan of 200 s (or, in some few slow cases, up to 1 hour).

### (a) Response to Transient-Localized Stimulus

As shown in Figure 2a, all four models respond to a single localized stimulus (Equations 8 and 9, indicated with dotted lines on the left domain edge, magnified 10 times for visibility). The WP and LEGI models respond most rapidly for the given parameter values, but the latter loses polarity as soon as the transient stimulus is turned off. The two Turing instability models, GOR and OT, take far longer to polarize: the amplitude of the peak in GOR continues to increase throughout the simulation, and OT only reaches a steady state amplitude by 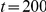 s, whereas the wave-based WP polarizes by 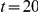 s. Both WP and GOR respond with a single polarized peak, whereas OT first develops multiple peaks and only later resolves these into a single front. Note that for the parameter sets we used, the highest amplification is exhibited by the two Turing-type models.

### (b) Response to Two Transient Stimuli at Opposite Cell Edges

As shown in Figure 2b, all four models develop a transient period with two responding peaks of activity mirroring the double stimulus. As before, LEGI returns to baseline as soon as the stimuli are removed. Of the remaining three models, all eventually have a single winning peak of frontness. However, the timescale on which stimuli are resolved is very long (thousands of seconds). Note that when the secondary peak collapses, the WP model responds by broadening the remaining peak without much of a change in the amplitude, while the Turing models respond by increasing the peak amplitude, while maintaining the width of the peak. If one of the stimuli is larger in magnitude, then the resolution of the stimuli is accelerated in all models (not shown), and the largest stimulus always takes over.

### (c) Response to Persistent Graded Stimulus

When a transient gradient is used as stimulus, results (not shown) are similar to a transient-localized stimulus. We next compare the responses of the models to persistent gradients of various steepness (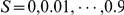 in Equation 10). Responses at 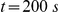 are shown in Figure 2c. Several differences are noteworthy. The WP model exhibits a switch-like response; unlike both Turing-type models, it requires gradients larger than some threshold to respond. The LEGI model responds in a way that increases with stimulus strength. The OT model shows the same response regardless of gradient steepness. GOR model responds to stimuli of all steepnesses, but polarizes faster for stronger stimuli.

### (d) Response to Gradient Reversal

As shown in Figure 2d, neither GOR nor OT are able to respond to a reversal of the stimulus direction (stimuli as in Equations 11–13). Both WP and LEGI are able to reorient. However, for WP, the new stimulus has to be larger than a certain threshold, while no such requirement exists for LEGI.

### (e) Response to Noise

In keeping with the above, all models other than LEGI respond to noise by producing a chemical pattern (Figure 2e). Unlike GOR and OT, the WP model returns to baseline if the noise amplitude fails to exceed a threshold (results not shown). Both GOR and OT respond to noise at an arbitrarily low level. In the case of OT, the pattern formed has multiple peaks of activity, and does not correspond to a polar pattern, but resolves on a longer timescale (similar to Figure 2b). WP can either form a polar pattern, as shown in the figure, or form multiple peaks that are resolved on a longer timescale.

### (f) Effect of Cell Size

All our previous calculations were done for a cell of size 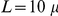
m. To test the behavior of the models for cells of various sizes, note that if we rescale space to 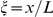, diffusion coefficients get rescaled to 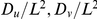. (Therefore, increasing the domain size is equivalent to decreasing the diffusion coefficients.) We examine the effect of a series of domain sizes (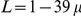
m) on the models' ability to resolve multiple peaks in Figure 2f. Models WP, GOR, and OT were stimulated with two transient stimuli (as in Figure 2b) and then compared at 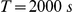 (approximately 30 min) after initial simulation. (For ease of comparison, homogeneous [nonpolar] solutions are indicated with a dotted line, and multi-peak solutions with a dashed line.) We find that a minimum size is required for polarity to emerge. The smallest “cells” (or cell fragments) of size 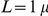
m fail to polarize in all three pattern formation models (WP, GOR, and OT). The GOR model produces a unique peak that concentrates all of the polarity regulator. Since the total amount of material in the simulation increases with cell size, the amplitude of the peak increases as the cell gets larger. Larger cells take longer to reach steady state. (Note that the location of the peak is still shifting at 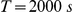 for large 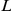.) The OT model generally responds by initially forming multiple peaks and resolving them over time. Again, larger cells take longer to reach steady state, hence at 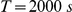 we still observe a quasi-stationary two-peak state for large 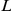. By contrast, the WP model responds with a single peak for 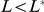, and two persistent peaks for 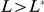. The exact value of this critical size 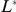 depends on the total amount of material in the system. In summary, all models predict that larger cells should take longer to resolve two competing stimuli, but eventually a single front will emerge. The timescale on which a single winning peak appears depends on the relative strengths of the stimuli (not shown).

We used persistent stimuli for the LEGI model as the system returns to basal unstimulated cell for transient stimuli. Domain size does not affect the LEGI model, as the activator and inhibitor profile simply mirror the profile of the external signal.

### Effect of Parameter Variation

The above simulations for the four models were carried out with parameter values as published in the cited sources. To explore robustness of these models to parameter variations, we also ran tests with a 1.5-fold increase or decrease in the values of certain key model parameters.

Results of such parameter variations can be summarized as follows: (1) In the GOR model, varying the strength of feedback 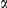 or basal activation rate 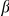 up or down by 1.5-fold has an imperceptible effect. Changing the initial amount of 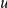 slightly alters the height of the single peak of activity, whereas the turnover rate 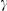 has a much more pronounced effect: increasing 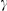 more than doubles the peak height of 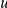. (2) The OT model is insensitive to variation of the basal activation rate 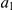, and only mildly affected by variation of the initial amount of 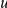 (slight increase/decrease of peak height). It is highly sensitive to both parameters 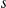 and 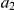, with a failure to polarize at one extreme of the parameter range (downregulation) and the formation of two peaks of activity at the other extreme (upregulation). (As discussed earlier, this is resolved into a single peak on a longer timescale.) (3) The LEGI model is unaffected by the production rate of the inhibitor 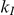, and only mildly affected by the initial amount of response element 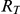, the production rate of response element 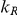, and the production rate of the activator 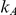. (These merely shift the profile up or down by a small value, but otherwise have little influence.) (4) In the WP model, increasing the initial amount of 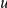 (which also increases the total amount of protein in the domain) shifts the wave of polarization further into the domain without affecting its amplitude. Varying the basal activation rate 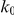 affects both front position and amplitudes of the plateaus. The WP model is more sensitive to the turnover rate 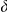, and feedback half-level 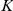
—at the 1.5-fold change (up or down) of either of these, the polarization is lost and the result is a spatially homogeneous state.

Overall, the WP model appears to be the only one in which the width of the polarized region (when it exists) undergoes dramatic change through up/down regulation of the total amount of protein. In contrast, varying the total amount of material for the other models mainly resulted in shifting the amplitude of the peak (or the rate of polarization), while the width of the peak remained the same. This forms one prediction that is amenable to experimental testing.

It is difficult to draw strong conclusions about robustness from this comparison of models, since some use nonlinear production rates, and other nonlinear degradation rates. In general, we found that GOR and LEGI were least responsive to parameter variations in the given range. WP and OT were most sensitive, occasionally exhibiting a homogeneous steady state (WP) or a secondary peak (OT) as described above.

### Contact between Experiment and Theory

It has often been the case that mathematical models for polarity were developed independently from the experiments and then demonstrated to reproduce previously obtained experimental results. Can mathematical models do more than just recapitulate experimentally observed behavior? Here, we mention examples of mathematical modeling that have fostered biological experiments. Gradient-sensing models such as LEGI have initiated experimental work on inhibitory mechanisms on the PI3K activation pathway in *D. discoideum* [87]. The inhibitory mechanisms found by that study were local rather than global in nature, and a global inhibitor is still unconfirmed. The multipipette experiments on immobilized *D. discoideum*, where cells are presented with two competing stimuli and showed two responses [22], were inspired by the LEGI model. However, it would be interesting to repeat these experiments in cells with an intact actin cytoskeleton and compare to the results obtained in Figure 2. In Goryachev's model for yeast polarization, a unique polarity axis emerges due to competition for substrate between clusters [54]. This inspired experiments to “re-wire” the positive feedback loop to get cells with multiple Cdc42 caps that then get resolved into a single bud [59]. Although these experiments are consistent with the idea that competition for a limited resource can resolve multiple foci, the details of how this competition is resolved on a tightly controlled timescale (a few minutes in the experiments, but longer in the model), or why a more intense cap does not always win, are still to be resolved.

In budding yeast, experiments have confirmed that the removal of one of the two parallel Cdc42 positive feedback loops does not abolish polarity [14], [88]. Interestingly, models of yeast polarization that utilize a single feedback loop predict different outcomes, depending on which loop is active. A deterministic model of positive feedback via actin-dependent transport predicts that increasing the total amount of Cdc42 concentration will lead to increased frequency of polarization, while a stochastic model for actin-independent (diffusion-mediated) positive feedback predicts a decrease in polarization frequency [56], [79]. No spatial model combining both of these positive feedback loops has been published, although some numerical simulations of spatially homogeneous systems suggest that a combination of fast and slow positive feedback loops can reduce the effect of noisy input and extend the parameter range for bistability [89].

In neutrophils, it has been suggested that microtubules allow communication between the front and back [34], [90]. However, the mechanism for this long-range feedback between the front and back is still unclear, and a more detailed model is needed. Why microtubules are needed for polarity in some cells, but not others, is also not understood, and models of polarity can be used to elucidate this issue.

The effect of cell size and geometry on signaling networks is just starting to be appreciated. For example, a simple model of membrane activation/cytoplasmic deactivation predicts that Cdc42 activity would be greatest where the cell is thinnest (i.e., lamellipodia and filopodia) [91]. All the models discussed so far in this review have been done on a 1-D domain and do not take into account nonuniform thickness or the shape of the cell. Simulations in 2-D reveal that the dynamic shape change of a cell can accelerate the process of polarization (S. Marée, personal communication). This speaks to studying the phenomena in 2-D and 3-D, rather than in 1-D domains only. The effect of biophysical parameters such as membrane tension has also been neglected in models of cell polarity. However, this is known to influence polarity regulators. For example, stretching a fibroblast along one axis inhibits Rac activity in the plasma membrane parallel to the direction of stretch, confining formation of new actin polymers to unstretched membrane domains [92].

New experimental tools allow for spatial manipulation of signaling on a subcellular level not possible earlier, such as activating a single polarity protein in a localized area [93]–[95]. These techniques should allow for experimental testing of the predictions of the many competing models of cell polarization.

## Discussion

It is not possible in a single review to be comprehensive, as the literature on polarity models has become quite large. Here, we subjected a limited set of (four) deterministic models for cell polarity to a number of standard stimulation protocols so as to compare their responses (summary in Table 2). From Figure 2 and Table 2 we see that models with a Turing instability generally have a lower sensitivity to second stimuli, and LEGI models have no inherent persistence. However, amplification is a common feature observed in all models, with “Turing-type models” exhibiting highest amplification in our hands. Note that the gradient-sensing models alone describe adaptation to uniform stimulus, but do not capture the phenomena of spontaneous polarization or polarity maintenance in the absence of stimulus. Wave-based and Turing models differ in their response to multiple stimuli and to a change in the direction of the stimulus. Thus, some classes of models are appropriate to describe some polarization behaviors but not others. Spontaneous polarization is described well by Turing instability models. Gradient-sensing models like LEGI seem most pertinent to cells without a cytoskeleton that do not exhibit maintenance or spontaneous polarity. Cells that need to rapidly reorient, such as neutrophils are best described by wave-based models.

What has emerged collectively from theoretical work so far? As yet, no one model or set of models have been “proven to be correct”, nor would we expect such proof in future. Every model is incomplete, and various models attempt, with different degrees of success, to capture some qualitative aspects. There are many complex processes in the cell, and, clearly, modeling focuses on some specific subsets and simplifies or ignores others. In interpreting the results of models or comparing with experiments such simplifications have to be borne in mind. Nevertheless, we can point to a number of significant paradigms resulting from theoretical work that help inform our intuition and understanding of the qualitative features of cell polarity.

(1) Amplification of weak stimuli and shallow gradients: all models point to the fact that some inherent pattern-formation mechanism is likely at play in cells to turn low-amplitude stimuli into macroscopic responses in polarized cells. This means that the mechanism should involve local activation and more long-ranged (“global”) suppression of activity [49], but whether the details involve actual global inhibitors, or depletion of inactive forms, or other more elaborate interaction networks with multiple local and global reach, is unclear. The fact that pattern-formation mechanisms involve some positive local influence and other negative long-range influences helps to explain an interesting aspect of polarity signaling systems: many of these, notably Rho GTPases, have both membrane-associated active forms and inactive cytosolic forms. The wide difference in mobility of these two forms facilitates the ability to form patterns, by inherently creating “local” (slow diffusing) and “global” (fast diffusing) forms. As predicted, the local forms are active, and the global forms inactive, in the case of Rho GTPases.

(2) Maintenance of polarity (unimodal pattern), once formed, is an automatic feature of most pattern-forming mechanisms, explaining the persistence of polarity even when stimuli are removed. As we have pointed out, this can create the issue of locking of a pattern, which is undesirable in cases where cells have to keep changing their polarity in response to a complex environment. (The WP mechanism described here does not have this problem.) To counteract this issue in Turing-type models, one could postulate that many underlying affinities or binding rates are constantly and rapidly changing (making the polar pattern destabilize) or that there is an underlying oscillation as described in [49]. Alternately, additional modules can react to change in gradient direction and “unfreeze” a pattern.

(3) Models that maintain polarity in the absence of a stimulus require positive feedback (see, e.g., Figure 1a–c, 1f). Adaptation models (Figure 1d, 1e) lack this feature. Autocatalysis of local activation is frequently associated with Turing instability. However, other behavior, such as WP, may also emerge from positive feedback. There is experimental evidence suggesting that feedback loops required for maintenance are provided by the cytoskeleton.

(4) The current models that account for spontaneous polarization do not seem to be compatible with cell adaptation. Again, this results from spontaneous polarity models assuming additional feedbacks that lead to maintenance of polarity rather than transient behavior of cell adaptation.

One must then be careful not to assume that even in the same cell type, the same mechanism of polarity establishment is at work in cells that polarize in a random direction, and in cells that adapt to a uniform gradient. As another example, a Turing-type model explaining the rise of spontaneous polarity in yeast may be appropriate when the resulting polar caps are stable (in the presence of actin cytoskeleton feedback), but it would not be appropriate to describe cells where that feedback is absent, and caps form and disappear.

(5) The formation of a single peak of activity (i.e., a unimodal rather than multi-peak pattern) requires careful tuning of parameters in some models. This leads one to question the robustness of such mechanisms in the face of cell diversity. (Why don't cells develop multiple stripes or spots of frontness?) In fact, some cells (such as *D. discoideum*) provide evidence of multiple and continually spawned pseudopods (“peaks of frontness”) [20], [83] and react to double-pipette stimuli with a double-faced response. Other cells resolve conflicts rapidly, owing in large part to additional layers of the signaling system that promote “winner takes all” competition between frontness peaks. (Recent work by A.F.M. Marée, personal communication, demonstrates that the phosphoinositide signaling by PIP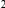 and PIP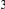 plays such a role via important positive feedback from a growing peak of GTPase activity to further growth of that peak.)

(6) Our review has focused on deterministic mechanisms of polarity establishment. However, in uniform or shallow gradients of chemoattractant, stochastic effects become important and noise reduction mechanisms are needed for accurate spatial sensing. For modeling insights on accurate gradient sensing from weak/noisy signals, see [96]–[98].

In short, the power of modeling lies in its abstraction. General schemes for cell polarity development have been proposed that, despite differences in specific molecular mechanisms, apply to many cell types. Here, we showed that each of the different proposed mechanisms for polarity regulates certain aspects of polarization behavior more strongly than others.

## Methods

Unless otherwise noted, model systems we focus on share the following basic (1-D reaction-diffusion) form
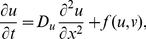
(1a)

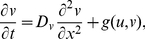
(1b)for 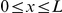. For all models, we used no-flux boundary conditions with domain size 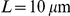 unless stated otherwise.

### WP Model

Here, in system (1), 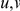 are the active, inactive forms of Rho GTPase, with 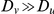 as discussed above, and
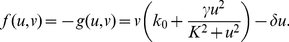
(2)The opposite signs for the reaction terms arise from mass conservation. For simulation of the WP model the following parameters were used: 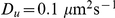, 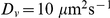, 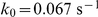, 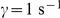, 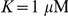, 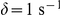. See [76] for justification of these values.

### OT Model

In the Otsuji model equations, [53], we employ (1) with
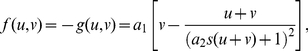
(3)with 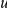 and 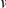 also representing active and inactive forms of Rho proteins, respectively.

To choose a set of parameters for this system that would permit a reasonable comparison of behaviors, we scaled the published parameters from [53] so that rates of diffusion match those of 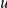 and 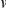 in the WP model (since both models consider Rho GTPases in similar cell types). Scaling time by a factor of 10 leads to the following parameter set: 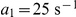, 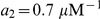, 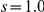, 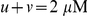, 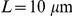, 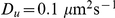, 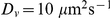. Finding the dispersion relation for system, we see that modes 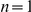 and 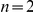 are unstable for these parameter values, so that both one and two peak patterns can occur.

### GOR Model

In Goryachev's reduced two-component model [54], the RD system (1) has
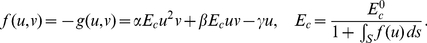
(4)Here, 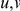 are GTP- and GDP-bound forms of Cdc42 and 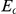 is the cytoplasmic Cdc24-Bem1 complex. The integral equation for 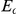 represents the conservation of total Cdc24-Bem1 complex. The diffusion coefficient for membrane-bound Cdc42 in yeast is significantly lower than in other cell types [54]. We used 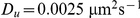, 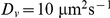, and calculated the reaction parameters 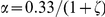, 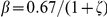, 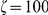, and 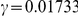 from the reaction rates given in [54]. Because of the much slower diffusion coefficient in this model, polarization takes much longer. For simplicity, 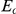 was taken to be a constant.

### LEGI Model

The LEGI model is given by
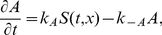
(5a)

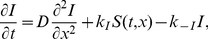
(5b)

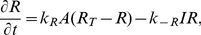
(5c)where 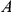 is the activator, 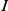 is the inhibitor, 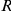 is the response element, and 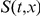 is the external stimulus [61]. We used 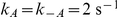, 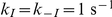, 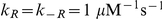, 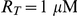, and diffusion coefficient 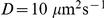. Adding a small diffusion coefficient 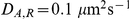 to the equations for activator and response element did not affect the results.

### Stimuli Repertoire

To test the effect of various stimuli on the WP, GOR, and OT models, we implemented
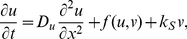
(6)

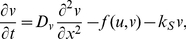
(7)where 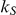 is a transient spatial signal, that is, a rate of activation of 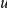, here considered to be the signaling agent. For the LEGI model, 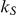 was used as the signal 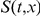 in Equation 5. The following set of stimuli were used for 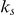:

- **Transient localized stimulus:** We stimulated 10% of the domain using
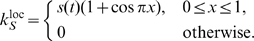
(8)where the time dependence was as follows:
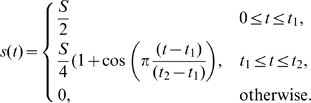
(9)We tested a range of parameters as follows: 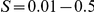, 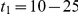 s, 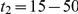 s.
- **Two transient localized stimuli:** We used the same stimulus as in (a) on 0%–10% of the domain, and a second stimulus of the same magnitude (reflected along the *x*-axis) on 90%–100% of the domain.
- **Persistent graded stimulus:**

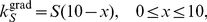
(10)where 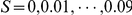.
- **Reversal of graded stimulus:** We applied
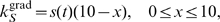
(11)where
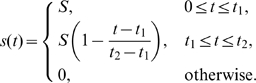
(12)followed by
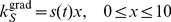
(13)where
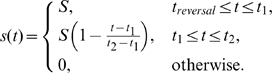
(14)

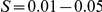
, 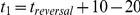 s, 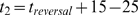 s were tested.
- **Random initial conditions:** We used nonuniform initial conditions with zero mean about the homogeneous steady state 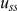 for all models. (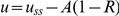, where 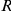 is a random number with uniform distribution in 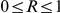.) Noise amplitude 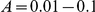 was used.
- **Effect of cell size:** Calculations were done on a unit size domain with diffusion coefficients 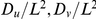 for 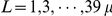
m. The same stimulus was used as in (b). Results at 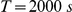 are shown.
